# Antimicrobial susceptibility testing to evaluate minimum inhibitory concentration values of clinically relevant antibiotics

**DOI:** 10.1016/j.xpro.2023.102512

**Published:** 2023-08-10

**Authors:** Lucien Barnes V, Douglas M. Heithoff, Scott P. Mahan, John K. House, Michael J. Mahan

**Affiliations:** 1 Department of Molecular, Cellular, and Developmental Biology, University of California, Santa Barbara, CA 93106, USA; 2 Department of Medical Microbiology and Immunology, School of Medicine, University of California, Davis, CA 95616, USA; 3 Faculty of Science, School of Veterinary Science, The University of Sydney, Camden, NSW 2570, Australia

**Keywords:** Health Sciences, Clinical Protocol, Microbiology

## Abstract

Antimicrobial susceptibility testing is used to determine the minimum inhibitory concentration (MIC), the standard measurement of antibiotic activity. Here, we present a protocol for evaluating MIC values of clinically relevant antibiotics against bacterial isolates cultured in standard bacteriologic medium and in mammalian cell culture medium. We describe steps for pathogen identification, culturing bacteria, preparing MIC plates, MIC assay incubation, and determining MIC. This protocol can potentially optimize the use of existing antibiotics while enhancing efforts to discover new ones.

For complete details on the use and execution of this protocol, please refer to Heithoff et al.^1^

## Highlights

• Protocol for antibiotic tests in cell culture medium to improve diagnostic accuracy
• Step-by-step guide for testing in cell culture medium
• Step-by-step guide for testing in human sera and urine

## Abstract (editor highlights)

Publisher’s note: Undertaking any experimental protocol requires adherence to local institutional guidelines for laboratory safety and ethics.

## Graphical abstract

**Figure:**
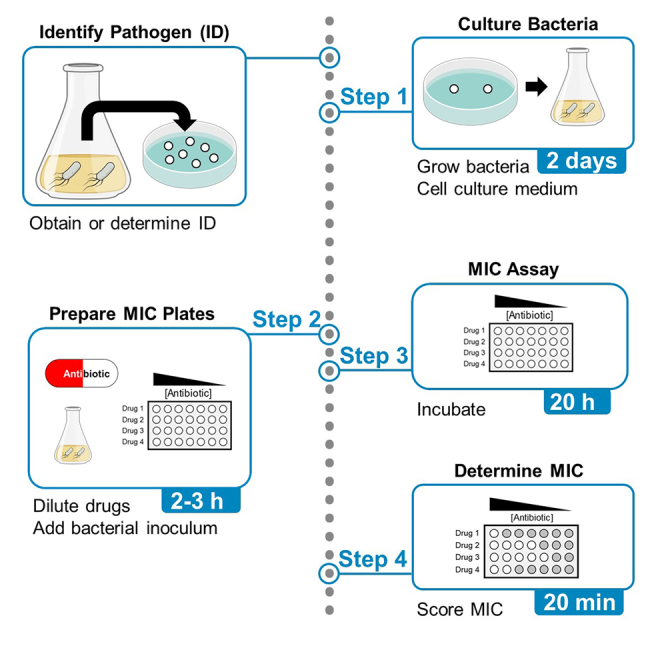

## Before you begin

Antimicrobial resistance (AMR) to existing medications is one of the biggest challenges facing public healthcare.^2^ Antimicrobial susceptibility testing (AST) is used to determine the minimum inhibitory concentration (MIC), the standard measurement of antibiotic activity. MICs define the clinical breakpoint, the concentration of antibiotic used to indicate whether an infection with a particular bacterial isolate is likely to be treatable in a patient. Clinical breakpoints are used by clinical microbiological laboratories to define patient isolates as susceptible (S), intermediate (I), or resistant (R) to a panel of antibiotics. Thus, the MIC assay is the gold standard for guiding physician treatment practices.

This protocol evaluates MIC values of clinically relevant antibiotics against bacterial isolates cultured in standard bacteriologic medium (cation-adjusted Mueller-Hinton broth [CAMHB]) and in mammalian cell culture medium (Dulbecco’s modified Eagle’s medium [DMEM]).^1^^,^^3^^,^^4^ Before commencing AST, it is essential to prepare the required reagents (media, buffers, antibiotics) (Table 1); identify the pathogen to be tested; select antibiotic concentration test ranges; and determine pathogen growth conditions to obtain adequate densities for reliable MIC determination.

**Figure 1:**
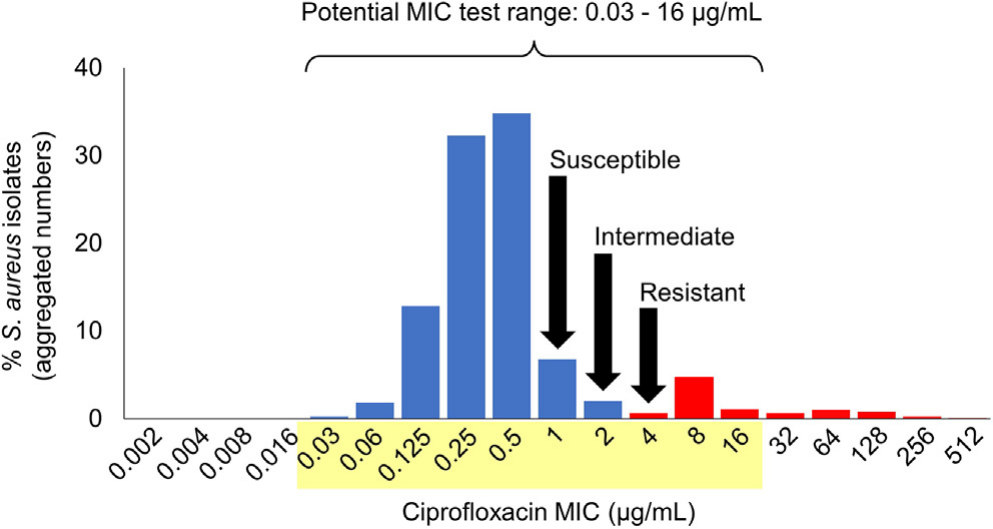
Antibiotic test range EUCAST curates a database of MIC results for a variety of antibiotics and bacterial pathogens that can be used to select an appropriate drug concentration range.^5^ Depicted is the MIC test range of ciprofloxacin against *S. aureus* (yellow), whereby the blue bars depict the percentage of *S. aureus* isolates classified as susceptible “S” or intermediate “I”; and red bars depict the percentage of isolates classified as resistant “R”.
    iv. Select a continuous range of ten 2-fold dilutions that encompass the clinical breakpoints used to categorize bacterial isolates as susceptible (S) or resistant (R) (if available) using the CLSI^6^ and EUCAST^7^ databases; e.g., https://www.eucast.org/clinical_breakpoints (Figures 1 and 2).

1. Identify Pathogen (ID).
  a. Obtain ID from clinical laboratory or determine by standard ID methods (PCR, microarray, immunology).
2. Determine antibiotic panel and concentration ranges.
  a. Select antibiotics with guidance from Clinical and Laboratory Standards Institute (CLSI) and European Committee on Antimicrobial Susceptibility Testing (EUCAST), or institutional policy.
  b. Determine MIC test range.
    i. Access the EUCAST MIC distribution repository^5^; https://mic.eucast.org/search/.
    ii. Select pathogen from the drop-down list.
    iii. Select antibiotic and view predicted susceptibility profile for each pathogen (e.g., *Staphylococcus aureus* susceptibility to ciprofloxacin) (Figure 1).

**Figure 2:**
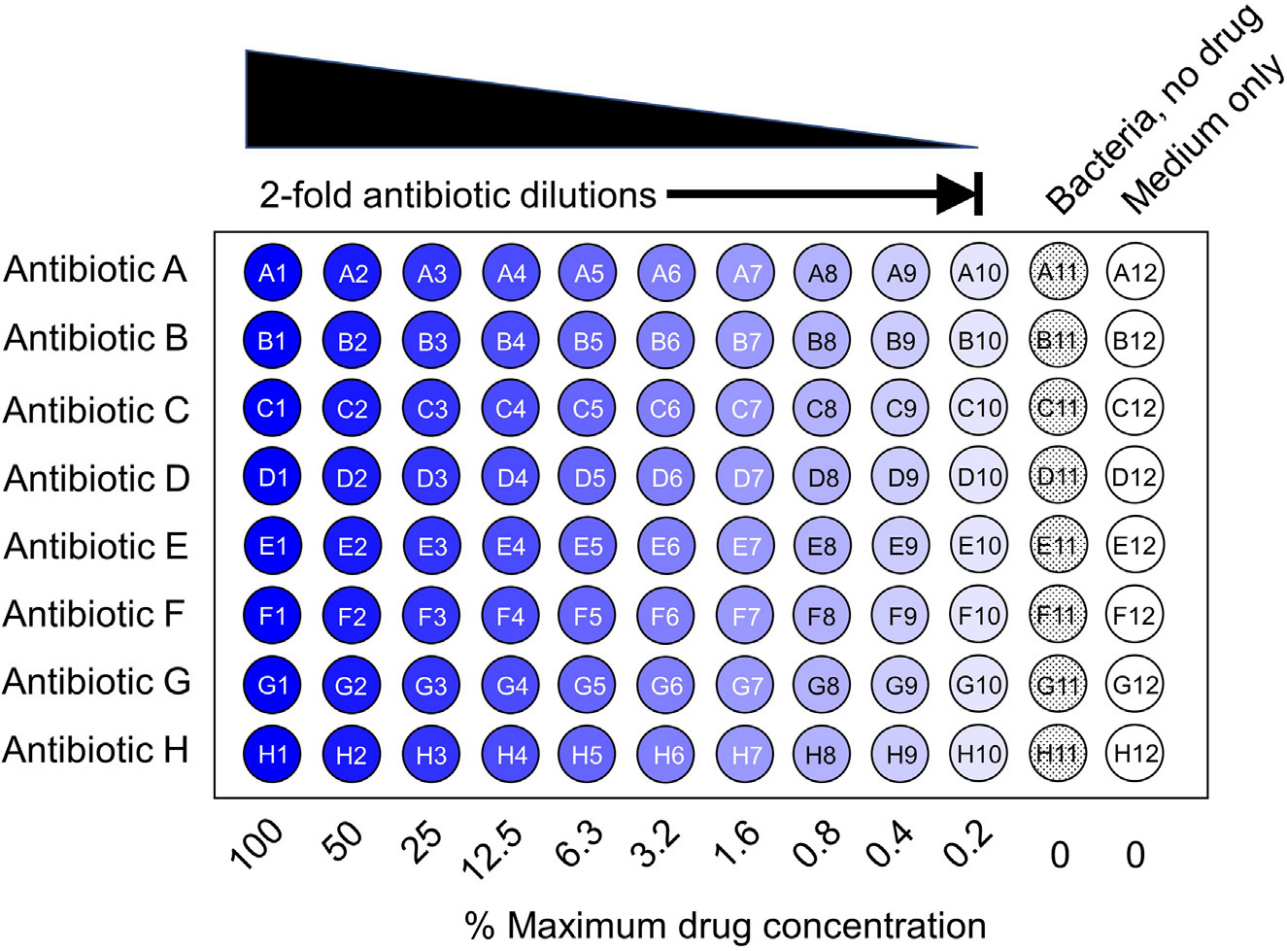
MIC schema Standard 96-well microtiter plates for MIC testing can accommodate eight antibiotics (A-H) and ten antibiotic concentrations, representing 2-fold drug dilutions of the maximum drug concentration tested (columns 1-10). The positive control wells contain bacteria without drugs (column 11). The negative control wells contain media only (column 12).
    v. Calculate 2 × the highest antibiotic concentration within the desired test range for each antibiotic (source for microtiter plate serial dilution).

***Note:*** Standard 96-well microdilution plates accommodate eight antibiotics for MIC testing: (8 antibiotics) × ([10 antibiotic concentrations] + 1 [positive control (bacteria, no antibiotic)] + 1 [negative control (media only)]).

3. Prepare antibiotic stock solutions.
  a. Antibiotic stock solutions are typically solubilized in deionized H_2_O (filtered and autoclaved) (10 mg/mL); vortex, and/or heat to 37°C (Table 1). If antibiotic is not soluble in H_2_O, use the least toxic solvent available (ethanol, methanol, acetone); optimal drug stock concentration > 1 mg/mL.
  b. Store at 4°C protected from light for up to two weeks. If antimicrobials are unstable at 4°C, store frozen as per manufacturer recommendations.
4. Determine bacteria concentration in standard and physiologic medium after 18 h culture (3 biological replicates).
  a. Culture bacterium (18 h) in standard (CAMHB) and physiologic media (DMEM).
    i. See step-by-step method details, Step 1, 2.
  b. Calculate bacteria concentration after culture.
    i. Serially dilute bacterial culture 1:10; repeat 5–7 times; plate 100 μL of last 3 dilutions on bacteriological media (step-by-step method details, Step 1).
    ii. Count colonies after 18 h incubation.
    iii. Calculate colony forming units (cfu/mL) according to the dilution factor (avg. of 3 replicates).

***Note:*** Alternatively, OD_600_ can be used to estimate cfu/mL; however, cfu/mL equivalents can vary between and within bacterial species.

**CRITICAL:** Human pathogen isolates are potentially hazardous. Always follow universal safety precautions and institutional guidelines while handing these materials.

**Table 1:** Commonly used antibiotic stock concentrations and solvents

Antibiotic	Type	Solvent^a^	Stock conc. (mg/mL)
Ampicillin	Ampicillin sodium	H_2_O	10
Azithromycin	Azithromycin dihydrate	Ethanol (∼95%)	10
Ceftriaxone	Ceftriaxone disodium salt	H_2_O	1
Cephalexin	Cephalexin monohydrate	H_2_O	10
Ciprofloxacin	Ciprofloxacin	0.1 N HCl	1
Colistin	Colistin sulfate	H_2_O	10
Daptomycin	Daptomycin	H_2_O	10
Ertapenem^b^	Ertapenem sodium	H_2_O	10
Imipenem^b^	Imipenem monohydrate	H_2_O	1
Linezolid	Linezolid	H_2_O	1
Piperacillin^c^	Piperacillin monohydrate	Methanol	10
Streptomycin	Streptomycin sulfate	H_2_O	10
Sulfamethoxazole	Sulfamethoxazole	Acetone	50
Tazobactam	Tazobactam	H_2_O	1
Tetracycline	Tetracycline hydrochloride	Methanol	10
Trimethoprim	Trimethoprim	Methanol	1
Vancomycin	Vancomycin hydrochloride	H_2_O	10

a H_2_O: deionized water (filtered and autoclaved).

b Store at -80°C (drug powder and subaliquots); store all other antibiotic subaliquots at 4°C.

c Piperacillin solubilization requires agitation (3 min).

## Key resources table

**Table:** 

REAGENT or RESOURCE	SOURCE	IDENTIFIER
**Bacterial and virus strains**		

*Acinetobacter baumannii*	ATCC 19606	2208
*Enterobacter cloacae*	ATCC 13047	CDC 442-68
*Enterococcus faecium*	Heithoff et al.^1^	MT3336
*Escherichia coli*	ATCC 25922	(Migula) Castellani and Chalmers
*Klebsiella pneumoniae*	ATCC 13883	NCTC 9633
*K. pneumoniae*	Heithoff et al.^8^	CRE MT3325
*Pseudomonas aeruginosa*	ATCC 10145	(Schroeter) Migula
*Salmonella enterica* Typhimurium	ATCC 14028	CDC 6516-60
*Staphylococcus aureus*, MRSA	Diekema et al.^9^	CA-MRSA USA300
*S. aureus*, MRSA	Heithoff et al.^8^	MRSA MT3302
*S. aureus*, MSSA	Yang et al.^10^	MSSA Newman
*Streptococcus pneumoniae*	Lanie et al.^11^	D39 (ser. 2)
*S. pneumoniae*	Carter et al.^12^	Daw 25 (ser. 35C)

**Biological samples**		

Human donor sera	Millipore Sigma	Cat # S1-LITER
Human donor urine	Innovative Research	Cat # 50-203-6075

**Chemicals, peptides, and recombinant proteins**		

Ampicillin	Millipore Sigma	Cat # A9518
Azithromycin	Millipore Sigma	Cat # PHR-1088
Ceftriaxone	Millipore Sigma	Cat # C5793
Cephalexin	US Pharmacopeia	Cat # 1099008
Ciprofloxacin	Honeywell Fluka	Cat # 17850
Colistin sulfate	Millipore Sigma	Cat # C4461
Daptomycin	Tokyo Chemical Industry Co.	Cat # D4229
Ertapenem	Millipore Sigma	Cat # SML1238
Imipenem	US Pharmacopeia	Cat # 1337809
Linezolid	US Pharmacopeia	Cat # 1367561
Piperacillin monohydrate	US Pharmacopeia	Cat # 1541500
Streptomycin	Fisher Scientific	Cat # BP910
Sulfamethoxazole	Honeywell Fluka	Cat # S7507
Tazobactam	US Pharmacopeia	Cat # 1643383
Tetracycline	Fisher Scientific	Cat # BP912
Trimethoprim	Millipore Sigma	Cat # T7883
Vancomycin	Millipore Sigma	Cat # V8138
Columbia CNA agar with 5% sheep blood	Becton Dickinson	Cat # 221352
Dulbecco’s modified Eagle’s medium (DMEM, high glucose)	Life Technologies	Cat # 11965-092
Luria-Bertani broth (LB)	Davis et al.^13^	Davis et al.^13^
Lysed horse blood (LHB)	Lampire Biological Laboratories	Cat # 7233402
Cation-adjusted Mueller-Hinton broth (MHB)	CLSI^14^	CLSI^14^
Todd-Hewitt broth (THB)	Becton Dickinson	Cat # 249240
Tryptic soy broth (TSB)	Becton Dickinson	Cat # 211825
Yeast extract (YE)	Genesee Scientific	Cat # 20-254

**Other**		

Conical tubes, 50 mL	Corning	Cat # 352098
Microfuge tubes, 1.7 mL	Genesee Scientific	Cat # 22-281
Microtiter plates (96-well)	Genesee Scientific	Cat # 25-104
Petri dishes	Genesee Scientific	Cat # 32-107G

## Step-by-step method details

### Culture bacteria under physiologic conditions (DMEM)

**Timing: 2 days**

Environmental sensitization to physiologic conditions during bacterial culture and AST can have up to a 1000-fold effect on antibiotic susceptibility.^15^ Consequentially, physiologic conditions should be implemented for any standardized AST protocol for widespread clinical utility. Detailed below is an AST protocol whereby both bacterial culture and MIC assays are performed in standard CAMHB and in DMEM cell culture medium.

1. Isolate bacteria on bacteriologic agar media.
  a. LB: Gram-negative pathogens.
  b. Incubate 18 h, 37°C, ambient atmosphere.
    ***Note:*** Pathogen-specific media/incubation.^14^
    i. *E. faecium/S. aureus*: TSB, incubate 18 h, 37°C, ambient atmosphere.
    ii. *S. pneumoniae*: CNA + 5% sheep blood, incubate 18 h, 37°C, 5% CO_2_ atmosphere.
2. Culture bacterium (3 biological replicates).
  a. Inoculate 1 colony per replicate into 0.5 mL of 100% CAMHB and DMEM.
  b. Incubate 18 h, 37°C.
    i. CAMHB, ambient atmosphere, shaking (225 rpm).
    ii. DMEM, 5% CO_2_ atmosphere, standing.
      ***Note:*** Pathogen-specific media/incubation.^14^
    iii. *S. aureus* CAMHB: inoculate with 5–7 colonies; no incubation.
    iv. *S. aureus* DMEM: supplemented with 5% v/v LB; inoculate with 1 colony; incubate 18 h, 37°C, 5% CO_2_ atmosphere, standing.
    v. *S. pneumoniae* CAMHB: supplemented with 5% v/v LHB; inoculate with 5 colonies; incubate 4 h, 37°C; ambient atmosphere, standing.
    vi. *S. pneumoniae* DMEM: supplemented with 5% v/v LHB; inoculate with 5 colonies; incubate 4 h, 37°C; 5% CO_2_ atmosphere, standing.

### Prepare microtiter plates to determine MIC

**Timing: 2–3 h**

MIC testing requires preparing appropriate antibiotic concentration test ranges and bacterial inoculum concentrations for reliable MIC determination.

3. Prepare antibiotic dilutions (3 biological replicates).
  a. Prepare 100 mL of test media (CAMHB and DMEM).
  b. Prepare media-diluted drug stock.
    i. Dilute concentrated drug stock (e.g., 10 mg/mL) into ∼400 μL of test media (CAMHB or DMEM) to generate a media-diluted drug stock at 2 × the highest drug concentration in test range.
  c. Add 100 μL each media-diluted drug stock to wells in column 1 (rows A_1_-H_1_) on microtiter plate (Figure 2).
  d. Add 50 μL test media (CAMHB or DMEM) to columns 2 through 12.
  e. Serial dilution of antibiotics.
    i. Pipette 50 μL of antibiotic from wells in column 1 into column 2.
    ii. Pipette up and down 3 times; repeat serial dilutions from wells in columns 3 through 10.
    iii. Discard 50 μL from wells in column 10.
      ***Note:*** Pathogen-specific media.
    iv. *E. faecium* CAMHB/DMEM: supplemented with 30% v/v TSB.
    v. *S. aureus* DMEM: supplemented with 5% v/v LB.
    vi. *S. pneumoniae* CAMHB/DMEM: supplemented with 5% v/v LHB.^14^
4. Addition of bacterial inoculum.
  a. Dilute 18 h culture (Step 2b) to 10^6^ cfu/mL (2 × bacterial inoculum) in test media (CAMHB and DMEM). Seven mL of 2 × inoculum is required per microtiter plate.
    ***Note:*** Cfu/mL for each pathogen/media was already determined by direct colony count (see before you begin, Step 4).
    i. Transfer 100 μL to microfuge tube to verify inoculum cfu/mL in Step 6.
    ii. Decant remaining ∼7 mL to sterile Petri dish (to facilitate pipetting).
  b. Add 50 μL of 2 × bacterial inoculum to all wells except column 12 (media only).
  c. Add 50 μL of additional media to wells in column 12 (media only).
    ***Note:*** Pathogen-specific media.
    i. *E.faecium* CAMHB/DMEM: supplemented with 30% v/v TSB.
    ii. *S. aureus* DMEM: supplemented with 5% v/v LB.
    iii. *S.pneumoniae* CAMHB/DMEM: supplemented with 5% v/v LHB.^14^

### MIC assay incubation

**Timing: 20 h**

Constant incubation time is critical for reliable MIC determination.

5. Incubate 20 h, 37°C, standing.
  a. CAMHB, ambient atmosphere.
  b. DMEM, 5% CO_2_ atmosphere.
6. Confirm 2 × bacterial inoculum (10^6^ cfu/mL) (aliquoted in Step 4a).
  a. Serially dilute (∼10^3^-fold); plate 100 μL onto LB agar.
  b. Incubate 18 h, 37°C, ambient atmosphere.
  c. Verify actual 2 × bacterial inoculum is within 3-fold of target (10^6^ cfu/mL).
    ***Note:*** Pathogen-specific media.
    i. *E. faecium/S. aureus*: TSB agar; incubate 18 h, 37°C, ambient atmosphere.
    ii. *S. pneumoniae*: THB + 2% YE agar; incubate 18 h, 37°C, 5% CO_2_ atmosphere.

### Determine MIC

**Timing: 20 min**

The MIC is the lowest antibiotic concentration that inhibits bacterial growth.

**Figure 3:**
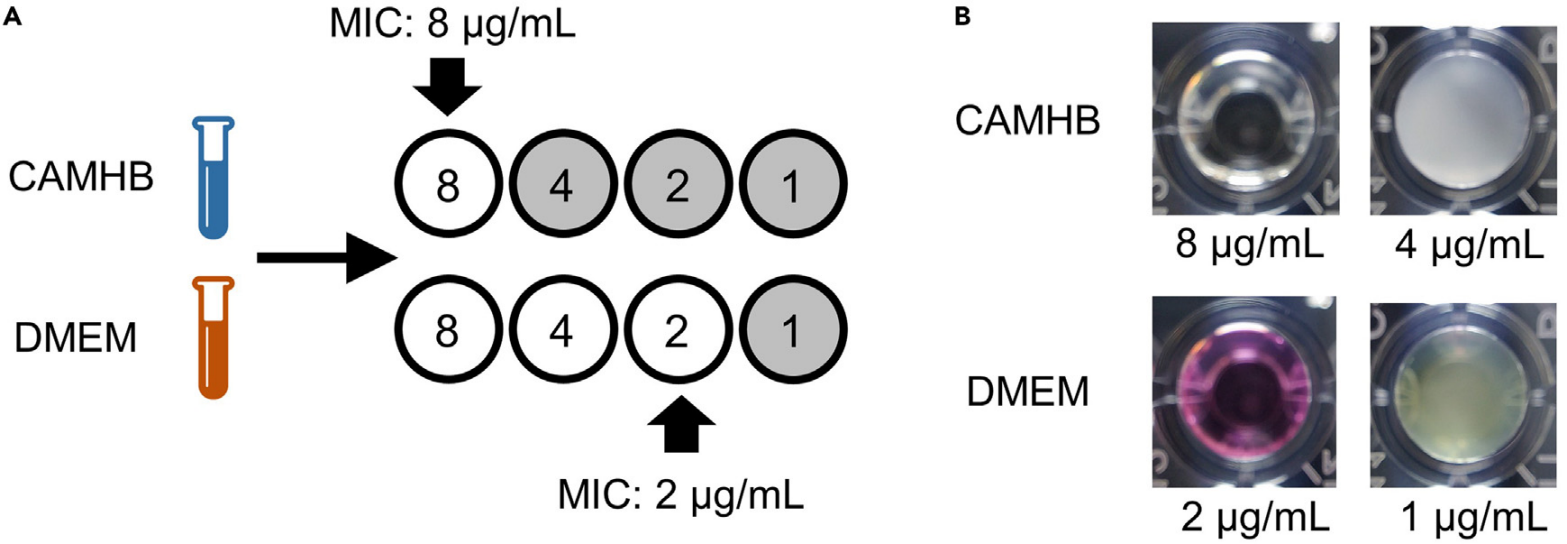
MIC determination (A) MIC assay performed on a bacterium grown in CAMHB and DMEM; gray circles depict bacterial growth within a microtiter plate well; white circles depict no growth. (B) Microtiter plate well images of an MIC assay with ertapenem tested against *S. aureus* grown in CAMHB (MIC = 8 μg/mL) and DMEM (MIC = 2 μg/mL).

7. Determine MIC.
  a. Score growth in test wells (presence/absence of turbidity) (Figures 3 and 4).

**Figure 4:**
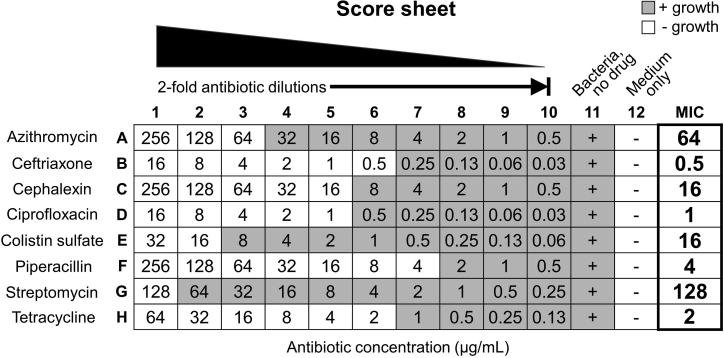
AST score-sheet Depicted is an exemplar AST score sheet of bacterial growth (gray) or no growth (white) in CAMHB as a function of antibiotic concentration on a microtiter plate (columns 1–10). The positive control wells contain bacteria, no drug (column 11). The negative control wells contain media only (column 12). The MIC is the lowest antibiotic concentration that inhibits bacterial growth and is recorded in the “MIC” column for each antibiotic (rows A–H).
  b. Confirm growth in bacteria/media, no drug wells (positive control).
  c. Confirm no growth in media-only wells (negative control).
8. Interpret MIC value with respect to clinical breakpoints.
  a. Susceptible (S), intermediate (I), or resistant (R) to antibiotics tested.

### Alternate AST protocol for human sera or urine

**Timing: same as CAMHB/DMEM protocol**

**Figure 5:**
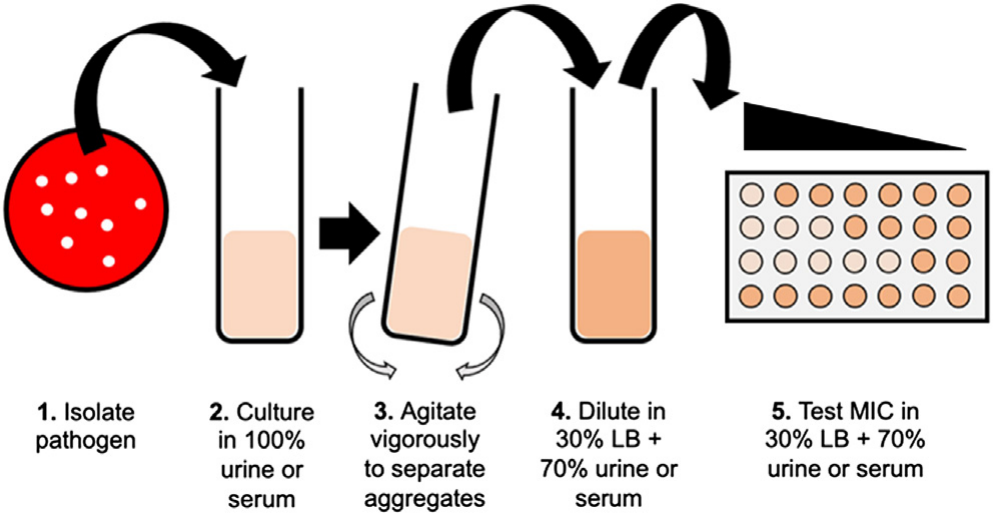
AST protocol for testing in pooled human donor sera or urine (1) Bacterial pathogens are isolated; (2) grown in 100% pooled human donor serum or urine; (3) agitated to separate bacterial cell-to-cell aggregates; (4) diluted into supplemented human fluids (30% LB + 70% sera or urine); and (5) MIC testing is performed in supplemented human fluids in microtiter plates.

AST in human sera and urine presents a formidable challenge as these host fluids can be inhibitory to bacterial culture. Some pathogens form bacterial cell-to-cell aggregates in sera and/or do not grow to adequate bacterial cell densities in sera or urine for reliable MIC determination. Detailed below is an AST protocol developed for pooled human donor sera or urine (Figure 5).

9. Isolate bacteria on bacteriologic media.
  a. Step 1, step-by-step method details.
10. Culture bacterium in undiluted pooled human donor sera or urine.
  a. Inoculate 1 colony/per replicate into 0.5 mL in host fluid (3 biological replicates).
  b. Incubate 18 h, 37°C.
    i. Sera: 5% CO_2_ atmosphere, standing.
    ii. Urine: ambient atmosphere, shaking (225 rpm).
      ***Note:*** Pathogen-specific media.
    iii. *A. baumannii*: heat-inactivated sera supplemented with 40% v/v CAMHB; heat-inactivate sera at 56°C for 30 min; mix (sera will form a thick gel at ∼60°C).
    iv. *S. pneumoniae* sera/urine: supplemented with 30% v/v THB, inoculate with 5 colonies; incubate 4 h, 37°C; sera: 5% CO_2_ atmosphere, standing; urine: ambient atmosphere, standing.
11. Prepare antibiotic dilutions.
  a. Step 3, step-by-step method details.
    ***Note:*** Pathogen-specific media.
    i. *A. baumannii*: heat-inactivated sera supplemented with 40% v/v CAMHB.
    ii. *E. faecium* sera/urine: supplemented with 30% v/v TSB.
    iii. *S. pneumoniae* sera/urine: supplemented with 30% v/v THB.
12. Addition of bacterial inoculum.
  a. Step 4, step-by-step method details.
    i. Vortex overnight inoculum (15 s, maximum speed) to disrupt aggregates.
  b. Dilute cultures to 2 × 10^6^ cfu/mL (2 × inoculum) in sera or urine supplemented with 30% v/v LB.
    i. Vortex (5 s, maximum speed, benchtop vortex) between dilutions.
  c. Transfer 100 μL to microfuge tube to verify actual 2 × bacterial inoculum is within 3-fold of target (2 × 10^6^ cfu/mL).
    i. cfu/mL verified in Step 14.
      ***Note:*** Pathogen-specific media.
    ii. *A. baumannii*: heat-inactivated sera supplemented with 40% v/v CAMHB.
    iii. *E. faecium* sera/urine: supplemented with 30% v/v TSB.
    iv. *S. pneumoniae* sera/urine: supplemented with 30% v/v THB.
13. Incubate 20 h, 37°C, standing.
  a. Sera: 5% CO_2_ atmosphere.
  b. Urine: ambient atmosphere.
14. Confirm 2 × bacterial inoculum (2 × 10^6^ cfu/mL).
  a. Aliquoted in Step 12.
  b. Step 4, 6, step-by-step method details.
15. Determine MIC.
  a. Step 7, step-by-step method details.
16. Interpret MIC value with respect to clinical breakpoints.
  a. Step 8, step-by-step method details.

## Expected outcomes

Clinical implementation of testing in cell culture medium may identify existing antibiotics for the potential treatment of AMR infections that are rejected by standard testing based on standard bacteriologic medium; and antibiotics that are ineffective despite indicated use by standard testing. Testing in DMEM revealed that β-lactam antibiotics were effective for the treatment of *S. aureus* in murine models of sepsis despite being rejected by testing in CAMHB (R to S, Table 2). Reciprocally, testing in DMEM revealed that colistin was ineffective for the treatment of *A. baumannii*, *K. pneumoniae*, or *P. aeruginosa* despite indicated use by testing in CAMHB (S to I/R). These data suggest that an AST experimental pipeline based on cell culture medium may improve the means by which antibiotics are tested, developed and prescribed. The protocol enables growth support for most bacterial isolates observed in clinical practice, and can be readily adapted to existing protocols and instrumentation. These features make the methodological transition to cell culture medium simple, scalable and affordable. Additionally, the experimental AST protocol based on human sera or urine has potential application for the translational development of precision personalized medicine that optimizes the identification and prescription of appropriate antibiotics for individual patients. Taken together, the experimental AST protocols described herein provide a platform for the discovery and development of new compounds as more accurate testing streamlines the identification of lead candidates early in the discovery process, potentially leading to significant time, cost and life savings.^1^

**Table 2:** Predictive accuracy of discordant MICs derived from AST in CAMHB vs. DMEM in Gram-positive and Gram-negative murine sepsis models^1^

	MIC values (μg/mL)		Mouse Survivors	CAMHB vs. DMEM Predicted/Actual
Pathogen/Antibiotic	CAMHB	DMEM		
Gram-positive				

MRSA USA300				
Ceftriaxone	256 R	8 S	10/10	R to S
Ertapenem	8 R	2 S	9/10	R to S
Piperacillin/Tazobactam	64/4 R	4/4 S	8/10	R to S
MRSA MT3302				
Ceftriaxone	64 R	8 S	8/10	R to S
Cephalexin	128 R	8 S	6/10	R to S
Piperacillin/Tazobactam	64/4 R	4/4 S	8/10	R to S
MSSA Newman				
Cephalexin	32 R	4 S	8/10	R to S

Gram-negative				

*A. baumannii* 19606				
Colistin	0.5 S	4 R	5/10	S to I/R
*E. cloacae* 13047				
Ceftriaxone	4 R	0.25 S	7/10	R to S
*K. pneumoniae* 13883				
Colistin	0.25 S	16 R	3/10	S to R
*K. pneumoniae* MT3325				
Tetracycline	4 S	16 R	5/10	S to I/R
*P. aeruginosa* 10145				
Colistin	0.5 S	8 R	2/10	S to R
*S.* Typhimurium 14028				
Streptomycin	16 I	4 S	9/10	I to S

MICs and susceptibility designations were determined by broth microdilution in CAMHB and DMEM.^16^^,^^17^^,^^18^Virulence assays: discordant MICs derived from AST in CAMHB and DMEM were tested for diagnostic accuracy in murine sepsis models (n = 10).^10^^,^^19^CAMHB vs. DMEM Predicted/Actual: the susceptibility designations denote the CAMHB predicted susceptibility vs. the DMEM predicted and actual clinical outcomes. S, susceptible; I, intermediate; R, resistant.

## Limitations

The AST experimental pipeline has the following limitations. First, MIC assays performed *in vitro* do not recapitulate all interactions between antibiotics and the host/bacterial pathogen, which can have a marked impact on drug potency. Second, results from the AST experimental pipeline cannot be generalized for MIC determinations within a species until a large number of clinical isolates are tested to ensure sufficient clinical representation. Third, clinical outcomes derived from systemic infection may not apply to localized infections (respiratory, skin, UTI) and thus, testing in physiologic media more representative of the corresponding site of infection might increase diagnostic accuracy. Last, the safety and efficacy of antibiotics identified by the experimental pipeline in animals must be confirmed in human studies before they can be generalized for patient treatment.

## Troubleshooting

### Problem 1

Insufficient bacterial growth during cell culture and/or MIC assay (Step 2, 7, step-by-step method details).

### Potential solution

• Media supplementation with rich media (LB, CAMHB or TSB) at 30% v/v.
• Increase supplemented above 30% v/v.

### Problem 2

All MIC-test wells containing bacteria and antibiotic are turbid (columns 1–10); or none of the MIC-test wells are turbid (columns 1–10) (Step 7, step-by-step method details).

### Potential solution

• MIC > highest drug concentration tested (all test wells are turbid); retest with higher drug concentration range.
• MIC < lowest drug concentration tested (none of the test wells are turbid); retest with lower drug concentration range.

### Problem 3

Inconsistent bacterial growth in sera during cell culture and/or MIC assay (Step 10, 15, alternate AST protocol for human sera or urine).

### Potential solution

• Increase vortex time to disrupt bacterial cell-to-cell aggregates.
• Minimize standing time before cell dilution series and bacterial plating.

## Resource availability

### Lead contact

Further information and requests for resources and reagents should be directed to the lead contact, Michael J. Mahan (mahan@ucsb.edu).

### Materials availability

This study did not generate new unique reagents.

## Data Availability

• All data reported in this paper will be shared by the lead contact upon request • This study did not generate new sequencing data or code. • Any additional information required to reanalyze the data reported in this paper is available from the lead contact upon request.
